# Potent Neutralization Antibodies Induced by a Recombinant Trimeric Spike Protein Vaccine Candidate Containing PIKA Adjuvant for COVID-19

**DOI:** 10.3390/vaccines9030296

**Published:** 2021-03-22

**Authors:** Jiao Tong, Chenxi Zhu, Hanyu Lai, Chunchao Feng, Dapeng Zhou

**Affiliations:** 1 Tongji University School of Medicine, Shanghai 200092, China; tongjiao2109@outlook.com (J.T.); chenxizhu614@hotmail.com (C.Z.); laihanyu@tongji.edu.cn (H.L.); chunchao0420@outlook.com (C.F.); 2 Shanghai Pudong New Area Mental Health Center Affiliated with Tongji University School of Medicine, 165 Sanlin Road, Shanghai 200124, China

**Keywords:** COVID-19, vaccine, neutralizing antibodies, adjuvant

## Abstract

The structures of immunogens that elicit the most potent neutralization antibodies to prevent COVID-19 infection are still under investigation. In this study, we tested the efficacy of a recombinant trimeric Spike protein containing polyI:C (PIKA) adjuvant in mice immunized by a 0–7–14 day schedule. The results showed that a Spike protein-specific antibody was induced at Day 21 with titer of above 50,000 on average, as measured by direct binding. The neutralizing titer was above 1000 on average, as determined by a pseudo-virus using monoclonal antibodies (40592-MM57 and 40591-MM43) with IC50 at 1 μg/mL as standards. The protein/peptide array-identified receptor-binding domain (RBD) was considered as immunodominant. No linear epitopes were found in the RBD, although several linear epitopes were found in the C-terminal domain right after the RBD and heptad repeat regions. Our study supports the efficacy of a recombinant trimeric Spike protein vaccine candidate for COVID-19 that is safe and ready for storage and distribution in developing countries.

## 1. Introduction

It is generally accepted that effective COVID-19 vaccines are the only approach to end the global pandemic. Currently, inactivated virus [1], adenoviral vector-based vaccines [2,3,4], and mRNA vaccines [5,6] encoding Spike proteins have been approved for urgent use in China, the US, and other countries. However, these vaccines do not meet the need for vaccination in all countries. For example, the inactivated COVID-19 vaccines are limited by manufacturing capacity due to difficulties in producing live viruses. The currently approved mRNA vaccines require cold-chain transport by freezers at −80 °C or −20 °C, although a thermostable mRNA vaccine candidate has been invented [7]. The other major unanswered question is the duration of immune responses induced by the above vaccines.

Vaccines based on recombinant proteins and adjuvants have been approved for HBV [8,9,10,11], HPV [12,13,14,15,16], and influenza [17,18]. Recombinant proteins containing novel adjuvants have shown higher efficacy compared to alum adjuvants. For example, HBV S antigens containing ODN 1178 induced significantly higher seroprotective responses, including those in hyporesponsive populations, compared with three doses of alum-adjuvanted Engerix-B [19,20,21,22]. The addition of the NKT cell agonist ABX196 to the HBV antigen resulted in protective anti-HB antibody responses in a majority of patients after a single injection [9]. The HPV vaccine Cervarix^TM^ (GSK, London, UK), containing the L1 protein of HPV-16 and HPV-18, and the Adjuvant System 04 (AS04) induces a high and sustained immune response against HPV [23]. Influenza antigens containing adjuvants have shown better protection and a low risk of side effects [24,25,26,27,28,29,30,31,32].

The manufacturing of recombinant proteins and adjuvants are easy to scale-up, which provides an unlimited supply of immunogens. Such vaccines have been proven to be safe and effective. More importantly, the immune responses often last for years. Thus, vaccines with the recombinant Spike protein and adjuvants as components may be among the best choices for developing countries to end the COVID-19 pandemic.

Both the full-length Spike protein and the engineered subunit of the Spike protein containing receptor-binding domain (RBD) have been reported as vaccine candidates [33,34,35,36,37,38,39,40,41,42,43,44,45,46]. The receptor-binding domain binding to the ACE2 receptor has been considered as the key target for vaccine development [33,34,35,36]. However, the neutralizing antibodies toward the N-terminal domain (NTD) alone have been found to be sufficient to be protective in COVID-19 convalescent plasma [47]. The neutralizing epitopes at NTD are important to protect against new COVID-19 variants with mutations at the RBD, such as B.1.351 and B.1.1.7 [48,49,50,51,52]. Vaccine candidates of the S1 subunit containing NTD have been tested [40,41], but whether the conformation of the monomeric S1 subunit is the same as that in the trimeric Spike protein is unknown. Both the RBD-Fc that contains the RBD dimer linked by the Fc region of immunoglobin [38,39], and the rationally designed RBD dimer based on the crystal structure [36], have been tested as vaccine candidates. Full-length Spike protein trimer vaccines have been developed for both native trimers [42] and trimers with prefusion-stabilized conformation [42,43,44,45].

In this study, we characterized a trimeric full-length Spike protein containing PIKA (polyI:C) adjuvant. We chose such a composition for several reasons: (1) the full-length Spike protein contains both RBD and non-RBD epitopes, as well as more T cell epitopes that are essential for inducing viral-specific T cells; (2) the conformation of the trimeric form of the Spike protein is similar to S-trimer structure in natural virions [42,53]; (3) the low-cost PIKA (polyI:C) adjuvant has shown excellent safety and efficacy in rabies vaccines by a 0–7–14 day vaccination schedule [54].

## 2. Methods

### 2.1. Expression and Purification of the Recombinant SARS-CoV-2 Spike Protein Trimer and Spike Protein Monomer

To express the prefusion Spike (S) ectodomain, a gene encoding the residues 1–1208 of 2019-nCoV S (GenBank: MN908947) with proline substitutions at residues 986 and 987, a “GSAS (amino acid sequence)” substitution at the furin cleavage site (residues 682–685), a C-terminal T4 fibritin trimerization motif, and an 8XHisTag were synthesized and cloned into the pcDNA3.1 vector. The plasmid was transfected to 293T cells and the recombinant S protein trimers were purified by nickel-nitrilotriacetic acid (Ni-NTA) chromatography (QIAGEN, Hilden, Germany), followed by size exclusion to further purify the trimers. The trimeric Spike protein was dissolved in a Tris-glycine native sample buffer (Thermo Fisher, Waltham, MA, USA), and separated by a 3%–8% Tris-acetate protein gel under a non-reducing condition. The trimeric Spike protein was visualized by a PAGE gel silver staining kit (Solarbio, Beijing, China). The monomeric Spike protein was produced as a secreted form by BTI-Tn-5B1-4 insect cells as described by the authors of [55].

### 2.2. Vaccination in Mice

Animal experiments were approved by the institutional board of Tongji University School of Medicine. A purified recombinant SARS-CoV-2 S-trimer-His tagged protein, or Spike protein monomer [55] was resuspended in PBS (pH 7.4) with PIKA adjuvant [54], TLR3 agonist provided by Yisheng Biopharma Ltd., Beijing, China). Groups of C57BL/6 mice (*n* = 5) at 6 to 8 weeks old were immunized by intramuscular injection of the PIKA-S-trimer vaccine or PIKA-S-monomer vaccine. Every mouse was immunized by 5 μg of S-trimer or S-monomer protein and 50 μg of PIKA adjuvant. Mice were immunized on Day 0, Day 7, and Day 14. Sera were collected on Day 21.

### 2.3. S-Protein-Specific Antibody Determined by ELISA

To measure the Spike-specific antibody, 96-well microplates (NUNC-Immuno, Thermo, Waltham, MA, USA) were coated by 50 μL 1 μg/mL S-trimer in PBS (pH 7.4) at 37 °C for 1 h and washed five times by 0.05% tween in PBS (PBS/T) on a mini-shaker. The plates were blocked by 1% bovine serum albumin (Sigma, St Louis, MO, USA) in PBS at 37 °C for 1 h and washed by PBS/T five times. Sera were diluted 25 times in the first well, followed by a 5-fold serial dilution completed 10 times. Sera were incubated at 37 °C with plate-bound S protein for 1 h and washed with PBS/T five times. Then goat anti-mouse IgG-conjugated HRP (Southern Biotech, Birmingham, Alabama, USA) was added with 5000-fold dilution in PBS, and incubated at 37 °C for 1 h. After washing five times, chromogenic substrates were added and incubated for 30 min. The reaction was stopped with H_2_SO_4_ solution (1M). The absorbance was measured at 450 nm and the antibody titer was calculated with Prism 7.0 (GraphPad Software Inc, San Diego, CA, USA).

### 2.4. Protein/Peptide Array

The protein/peptide array was performed as described [56]. Briefly, peptide-BSA conjugates, as well as S protein, S1 protein, RBD protein, and other proteins of SARS-CoV-2, were printed in triplicate on a PATH substrate slide (Grace Bio-Labs, Bend, OR, USA) to generate identical arrays in a 1 × 7 subarray format using a Super Marathon printer (Arrayjet, UK). The microarrays were stored at −80 °C until use. The arrays stored at −80 °C were warmed to room temperature and then incubated in a blocking buffer (3% BSA in PBS buffer with 0.1% Tween 20) for 3 h. A total of 400 μL of diluted sera or antibodies were incubated with each subarray for 2 h. The arrays were washed with PBST and the bound antibodies were detected by incubating them with Cy3-conjugated goat anti-mouse IgG (Jackson ImmunoResearch, West Grove PA, USA), and were then diluted at 1:1000 in PBST. The incubation was carried out at room temperature for 1 h. The microarrays were then washed with 1×PBST and dried by centrifugation at room temperature and scanned by LuxScan 10K-A (CapitalBio Corporation, Beijing, China) with the parameters set as 95% laser power/PMT 480. The fluorescent intensity was extracted by GenePix Pro 6.0 software (Molecular Devices, San Jose, CA, USA).

### 2.5. SARS-CoV-2 Pseudo-Virus Production

The extracellular domain of SARS-CoV-2 Spike protein (GenBank: MN908947) was engineered in a pcDNA3CMV-based-plasmid as a Zhou-COVID-19-Spike (Plasmid #161029, Addgene) to assemble a pseudo-virus more efficiently. Portions of VSV-G were used to replace the signal peptide and transmembrane region of SARS-CoV-2 Spike protein. The plasmids of 9 μg pHAGE-luciferase-GFP, 3 μg psPAX2, and 6 μg Zhou-COVID-19-Spike were co-transfected into HEK293T cells by mixing with 45 μL (1 mg/mL stock solution in H2O) polyetherimide (Polysciences, Warrington, PA, USA) and added into a DMEM medium containing 10% FBS. After 48 h and 72 h, the supernatant was harvested and pooled, respectively. The supernatant containing the pseudo-virus was centrifuged at 3000× *g* and filtered through a 0.45 μm sterilized membrane (Millipore, Burlington, MA, USA). The titer of the virus generated by the engineered Zhou-COVID-19-Spike plasmid was 10-fold higher than the non-engineered Spike protein sequence (data not shown), and remained stable after two rounds of freeze-thawing. The virus was stored in −80 °C as a culture supernatant and used directly for antibody neutralization assays without further purification.

### 2.6. Neutralization of Serum Antibody against Pseudo-Virus Infection

A 293T-ACE2 cell line (293T/ACE2) expressing human ACE2 was used for the virus neutralization assay. First, 3 × 10^4^ cells per well were seeded on 96-well plates 12 h before infection. Next, 50 μL of the pseudo-virus was incubated with an equal volume of serially diluted antibodies for 1 h at 37 °C. The monoclonal antibodies 40592-MM57 and 40591-MM43 (Sinobiological, Beijing, China) were tested as standards in parallel in the concentrations ranging from 0.1 μg/mL to 100 μg/mL. The mixtures of pseudo-viruses and antibodies were added to 293T/ACE2 cells. After 12 h of co-incubation, the co-culture medium was replaced with fresh DMEM containing 10% fetal bovine serum, and the samples were incubated for an additional 48 h at 37 °C. Luciferase substrate (Promega, Madison, WI, USA) was added at 100 μL per well to the lyse in the cells. The fluorescence was read by a microplate reader (TECAN). The 50% neutralization dose was calculated using Prism 7.0.

## 3. Results

### 3.1. Trimeric S Protein Induced Higher Neutralizing Antibodies

**Figure 1:**
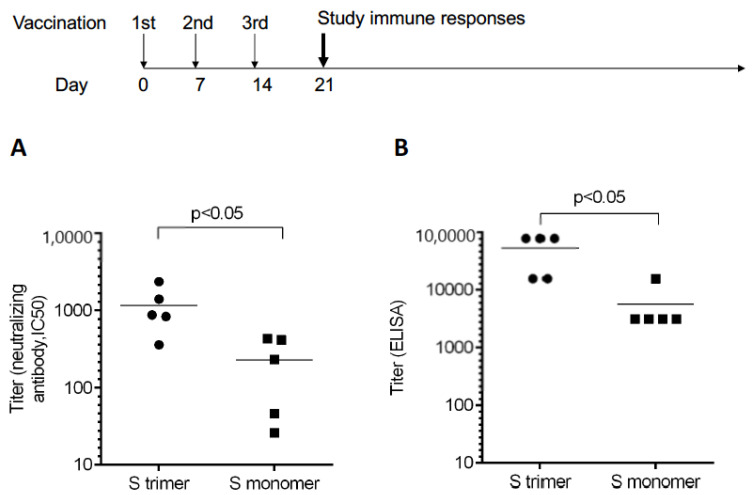
Antibody titers measured by neutralizing assays and ELISA. Mice were immunized by Spike trimer or monomer proteins containing PIKA adjuvant by a 0–7–14 day schedule. (**A**) The neutralizing titer as measured by pseudo-virus with monoclonal antibodies 40592-MM57 and 40591-MM43 as the control (with IC50 at 1 μg/mL). (**B**) Antibody titer as measured by ELISA using the plate-bound trimeric Spike protein. Data were representative of three independent experiments.

The titer of the antibody binding to the Spike trimer protein was above 50,000, on average, after three immunizations by trimeric Spike protein with polyI:C adjuvant. Using a pseudo-virus system, we determined the neutralizing titer to be higher than 1000 on average. Two monoclonal antibodies (40592-MM57 and 40591-MM43) with known neutralizing activities were used as standards for neutralization assays (with IC50 at 1 μg/mL). With the same dose of adjuvant and proteins, the trimeric Spike protein induced significantly higher neutralizing antibodies than the monomeric Spike protein (Figure 1).

### 3.2. Trimeric S Protein Induced Similar Epitope Patterns as Natural Infection

**Figure 2:**
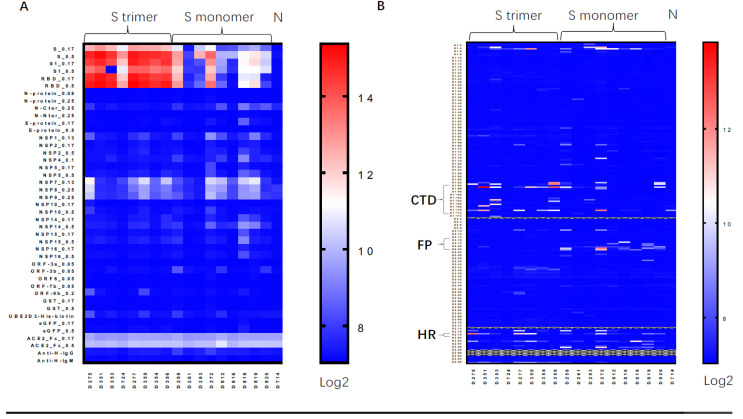
Protein/peptide array of serum antibodies induced by the Spike protein vaccines. (**A**) Protein-array assay for sera from mice immunized by the Spike trimer and Spike monomer; non-immunized mice using Spike (S_0.17 and S_0.5 mean proteins were printed at 0.17 or 0.5 mg/mL, respectively); the S1 subunit of Spike, the receptor-binding domain (RBD), and other viral proteins. (**B**) Linear peptide array using linear peptides of Spike proteins. C-terminal domain right after RBD (CTD; Peptides S1-93-S1-113); fusion peptide (FP; Peptides S2-14-S2-23); heptad regions (HR; Peptides S2-78).

To understand the epitopes of antibodies induced by the trimeric Spike protein vaccine, we performed a protein/peptide array containing a recombinant RBD, S1, and linear peptides of the Spike protein [56]. Serum from mice vaccinated by the trimeric Spike protein vaccine showed the strongest binding to the RBD, the S1 subunit, and the S proteins. However, linear epitopes were only observed in the C-terminal domain right after the RBD, and the heptad repeat regions (Figure 2). Few linear epitopes were found for the RBD region, indicating that the observed antibodies binding to the RBD region were non-linear confirmational epitopes. These results are highly consistent with the epitope patterns of serum from patients with natural infections of COVID-19 [56,57,58]. Our data support the hypothesis that the trimeric Spike protein induced antibody responses similar to natural virions.

## 4. Discussion

### 4.1. The Neutralizing Titer of Recombinant Trimeric Spike Protein Containing PIKA Adjuvant

The neutralizing titer of several versions of COVID-19 vaccines has been reported as ranging from 100 to 1000. Data on the standardized comparison of different vaccines are not available yet. In some studies, the level of the neutralizing titer from convalescent sera of COVID-19 patients was used as the control [59]. In our study, we used two commercially available monoclonal antibodies as the control in pseudo-virus-based assays and the neutralization titer was determined to be above 1000. In both the neutralization assay and direct binding to the Spike protein assay, the trimeric Spike protein showed significantly higher immunogenicity than the monomeric Spike protein (5-fold and 10-fold higher, respectively). This result is consistent with previous studies reporting that the prefusion-stabilized Spike trimer is most immunogenic, compared to the wild-type Spike trimer, S1 monomer and the RBD monomers [42]; although, the wild-type Spike trimer could induce a similar level of antibody titers as the prefusion-stabilized Spike trimer when used at a high dose (10 μg/mouse).

Table 1 summarizes the published COVID-19 candidates using recombinant protein technologies. The RBD can be expressed by a prokaryotic system and the immunogenicity of RBD can be maintained with no need for glycosylation [35]. The yeast expression system can efficiently glycosylate the Spike protein, but its efficiency in assembling trimeric Spike proteins remains unknown [33,34]. So far, the trimeric Spike proteins have been successfully expressed in insect cells, HEK293T and CHO cells as secreted proteins. The expression level of the secreted form of S-trimer in insect cells was reported as 1 mg/L for the wild-type S trimer and 5 mg/L for the prefusion-stabilized S trimer [42]. A recombinant membrane-anchored S trimer protein was produced by Novavax using recombinant baculovirus-infected Sf9 cells, and the S trimer was purified after lysing the insect cells with non-ionic detergent [43,60].

The high immunogenicity of the Spike trimer in its membrane form was previously reported in mRNA-based vaccines as prefusion-stabilized type [61], wild-type [62], or wild-type form with deleted furin cleavage site [62]. The deletion of the furin cleavage site caused higher stability of the membrane-anchored Spike trimer with increased binding to ACE2 and increased antigenicity [62].

### 4.2. The Neutralizing versus Binding Ratio of Vaccine-Induced Antibodies

The recombinant trimeric Spike protein with polyI:C adjuvant induced neutralizing antibodies with the titer higher than 1000, on average. Comparing this to the antibody titers induced by the mRNA vaccine, the inactivated virus, and the adenoviral vectors remains to be studied. According to previous studies by Walsh et al. [59], the ratio of neutralizing/binding of antibodies induced by the BNT162b2 mRNA vaccine is about 10-fold lower than antibodies from naturally infected patients (1:7 by natural infection as compared to 1:20 to 1:40 by vaccination). The mechanisms for a lower neutralizing/binding ratio might be due to the incomplete translation of mRNA, and/or the incorrect folding of the Spike protein. The neutralizing/binding ratio of antibodies induced by the trimeric Spike protein in human individuals will be evaluated in the near future when human subjects are vaccinated.

### 4.3. The Epitope Map of Antibody Induced by Vaccines versus Natural Infection

The similar antibody binding epitope map identified by the protein/peptide array suggested that the trimeric recombinant vaccine’s confirmation was similar to the Spike protein of natural virions. RBD was immune dominant, but few linear epitopes were found. The map of linear epitopes induced by the trimeric Spike protein were strikingly similar to natural infection. It is noteworthy that the monoclonal antibodies with neutralizing activities were identified to recognize the N-terminal domain NTD [63]. Poh et al. identified neutralizing antibodies that bind to the C-terminal domain right after RBD (CTD) and the fusion peptide (FP) region [57]. A structural analysis by Cryo-EM and molecular modeling indicated that the heptad repeat (HR) regions were surface-exposed and served as targets for neutralizing antibody binding [55,64,65]. Clearly, vaccines that can target the non-RBD region play an important role in preventing the pandemic of mutant viruses with RBD mutations that escape the RBD-focused vaccines.

### 4.4. The Efficacy of PIKA Adjuvant

PIKA adjuvant is a form of TLR3 agonist, which serves as a low-cost and highly effective adjuvant for vaccines [54,66,67,68,69,70,71]. When used as adjuvant for inactivated rabies vaccines in human subjects, the inactivated rabies vaccines containing PIKA induced a higher neutralizing titer [54]. In mice, the vaccine containing PIKA induced higher antibody titers than the vaccine containing an aluminum adjuvant [70]. Furthermore, in human studies using an accelerated vaccination schedule (two doses at Day 0, two doses at Day 3, and one dose at Day 7), 75% of recipients vaccinated by the inactivated rabies virus containing PIKA vaccines achieved a neutralizing titer above 0.5 IU/mL on Day 7, compared to 16.7% in the classic inactivated vaccine [71]. The safety profile of the PIKA rabies vaccine was non-inferior to the classic inactivated rabies vaccine. In future studies, we will test the efficacy and safety of the recombinant trimeric Spike protein containing PIKA by several vaccination schedules to establish a fast, durable, and protective immunity.

A highly competitive vaccine should be highly reliable to protect elderly people, who are most vulnerable to severe forms of the COVID-19 disease. In ongoing experiments on human vaccination studies, elderly people and those with predisposed conditions are the prioritized group to be recruited and analyzed.

## 5. Conclusions

Recombinant trimeric Spike protein containing PIKA adjuvant is highly immunogenic and induces potent neutralizing antibodies. Mechanistic studies suggest similar structures and conformation between a recombinant trimeric Spike protein vaccine and natural virions.

## Figures and Tables

**Table 1:** Recombinant proteins containing adjuvants for COVID-19.

Name of Vaccine	Developer	Protein or Subunit	Production	Adjuvant	Reference
RBD219-N1	Baylor College of Medicine	RBD	Yeast	Alum	[33,34]
SERS-S1	University of Pittsburgh School of Medicine	S1 subunit	HEK293K cells	MPLA	[41]
NVX-CoV2373	Novavax, Inc	Full-length trimeric Spike, protease resistant along with 2 proline substitutions at residues K986 and V987	Sf9 insect cells	Matrix-M (Quillaja saponins formulated with cholesterol and phospholipids into nanoparticles)	[43]
RBD-mFc	Guangzhou University of Chinese Medicine	RBD	Expi293 cells	Alum/Freund’s complete adjuvant	[38]
S-2P	Xiamen University	Secreted, prefusion stabilized trimeric Spike	Sf9 insect cells	Alum	[42]
RBD-Fc	University of Pittsburgh Medical School	RBD	Expi293	MF59 (squalene)	[39]
Spike S1	University of Hawaii	S1 subunit	HEK293T cells	CoVaccine HT™ (synthetic sucrose fatty acid sulphate esters (SFASES) immobilized inside the oily droplets of the submicron squalane in water emulsion)	[40]
S1-4	Chinese Center For Disease Control and Prevention	RBD	*E.coli.* BL21(DE3)	Alum	[35]
RBD-sc-dimer	University of Chinese Academy of Sciences	RBD-dimer protein	HEK293T cells	AddaVax (MF59)	[36]
S1, RBD	Chinese Academy of Medical Sciences and Peking Union Medical College	S1 subunit and RBD	HEK293T cells	Alum	[37]

## Data Availability

Not applicable.
